# Vesico-vaginal Fistula

**Published:** 1856-08

**Authors:** Frank H. Hamilton

**Affiliations:** Buffalo


### ART. V.— *Vesico-vaginal Fistula. Successful Operation.* By Frank H. Hamilton, M. D., Buffalo.

Mrs. ——, æt. 45. The fistula was produced nearly eight years ago, in her first labor. Forceps were employed.

The opening was at the usual place, just above the pubis, and about two inches from the vulva. Its shape was oval, with its longest diameter transverse. Its size was such as to receive easily the end of the index finger. At the upper end of the vagina a thin septum traversed the passage, leaving only a small opening through which the menses could escape. She had ceased menstruating nearly a year before.

I gave her chloroform, and, assisted by Dr. Eastman, I removed the edges of the fistula very freely. This part of the operation was made partly with strong scissors, but chiefly with a long bistoury. When the incisions were completed the opening was very much larger than before, and the abraded surfaces were extensive. When the bleeding had ceased we closed the wound with four strong, silk sutures, each suture being introduced free from the margin of the opening, and being made to penetrate deeply. Most of the sutures were introduced by the aid of a reversed needle, attached to a long handle.

During the operation the patient rested upon her back, but as soon as it was completed, she was laid upon her belly, and a short, flexible metallic catheter introduced, bent into the shape of the italic letter s.

During forty-eight hours this position, always a very tedious one, was steadily maintained, After this she was permitted to lay upon her side and even occasionally to sit up. On the seventh day three of the sutures were removed, and on the tenth the fourth and last was removed. On the same day, also, the catheter was discontinued.

The success of the operation has been complete, so that on the tenth day she passed her urine voluntarily, and retained it also without inconvenience. Not one drop has passed through the fistula since the operation was made.

